# Water Soluble Self-Aggregates Induced Green Emission of Biocompatible Citric Acid-PEG Hyper Branched Polymer

**DOI:** 10.1038/s41598-017-16683-w

**Published:** 2017-11-27

**Authors:** Gajendiran Mani, Kim Kyobum, Balasubramanian Sengottuvelan

**Affiliations:** 1 Department of Inorganic Chemistry, Guindy Campus, University of Madras, Chennai, 600025 India; 2 Division of Bioengineering, College of Life Sciences and Bioengineering, Incheon National University, Incheon, 22012 Korea; 3 Center for Advanced Materials Research, Vels University, Chennai, 600117 India

## Abstract

An aliphatic citric acid–PEG hyper-branched polymer (CPHP) with a π-bond on the polymer backbone was synthesized by a single- step melt reaction in which the polymerization and π-bond formation occur simultaneously. The chemical structure of CPHP was confirmed by FTIR, ^1^H-NMR, ^13^C-NMR and MALDI-TOF mass spectral analyses. Aggregates are generally found to disperse in any solvent but the CPHP aggregates were soluble in water due to their hybrid nature. The π-bond in the aconitate unit induces green emission by CH/π interaction while the PEG unit of CPHP increases its solubility in water. The soluble aggregates induced green emission (SAIE) of the CPHP was investigated by UV-Visible absorption and emission spectra, time- correlated single photon counting (TCSPC) and zeta potential measurements. The fluorescence life time (τ_f_) increased from 4.93 to11.38 ns with an increase in CPHP concentration. The fluorescence quantum yield (Φ_f_) of CPHP can be altered by varying the concentration of CPHP.

## Introduction

Synthesis of novel macromolecules is significant because of their versatile application in areas such as dye sensitized solar cells^1,2^, metal nanoparticle (NPs) synthesis^3,4^, bio-imaging^5,6^ and drug delivery^7^. Silver NPs (AgNPs) and gold NPs (AuNPs) are synthesized by using dendrimers such as poly (amidoamine) and polyphenylene^8,9^. The dendrimer-AuNP conjugates find extensive application in biomedical field^10^. Especially, synthesis of hybrid macromolecules such as citric acid (CA)–polyethylene glycol (PEG) based macromolecules and their potential applications in drug delivery have been reported^11–13^. PEG is used as a core unit while CA is used to build the backbone of the CA-PEG linear dendrimer with molecular weight below 3800 (g/mol)^14^.

Photo luminescence usually occurs in dilute solutions and its intensity decreases when increasing the concentration of luminophore due to self-quenching^15,16^. Interestingly, the opposite process to self-quenching called aggregation- induced emission (AIE) occurs in some organic compounds such as substituted biphenyl, silole molecules, tetraphenylethene-cored luminogen and cyclophanes^17–20^. Aggregates are usually formed by adding a non-solvent to a solution and finally the aggregated particles are in dispersed form. Aggregates which are in clear solution are known as “soluble aggregates” and proteins exhibit this behavior^21,22^. Recently AIE has been employed to detect the cancer cells^23^. In the present investigation, synthesis of a new type of aliphatic citric acid–PEG hyper- branched polymer (CPHP) with a π-bond on the polymer backbone and its water soluble aggregates- induced green emission (SAIE) behavior are reported for the first time.

## Results and Discussion

**Figure 1:**
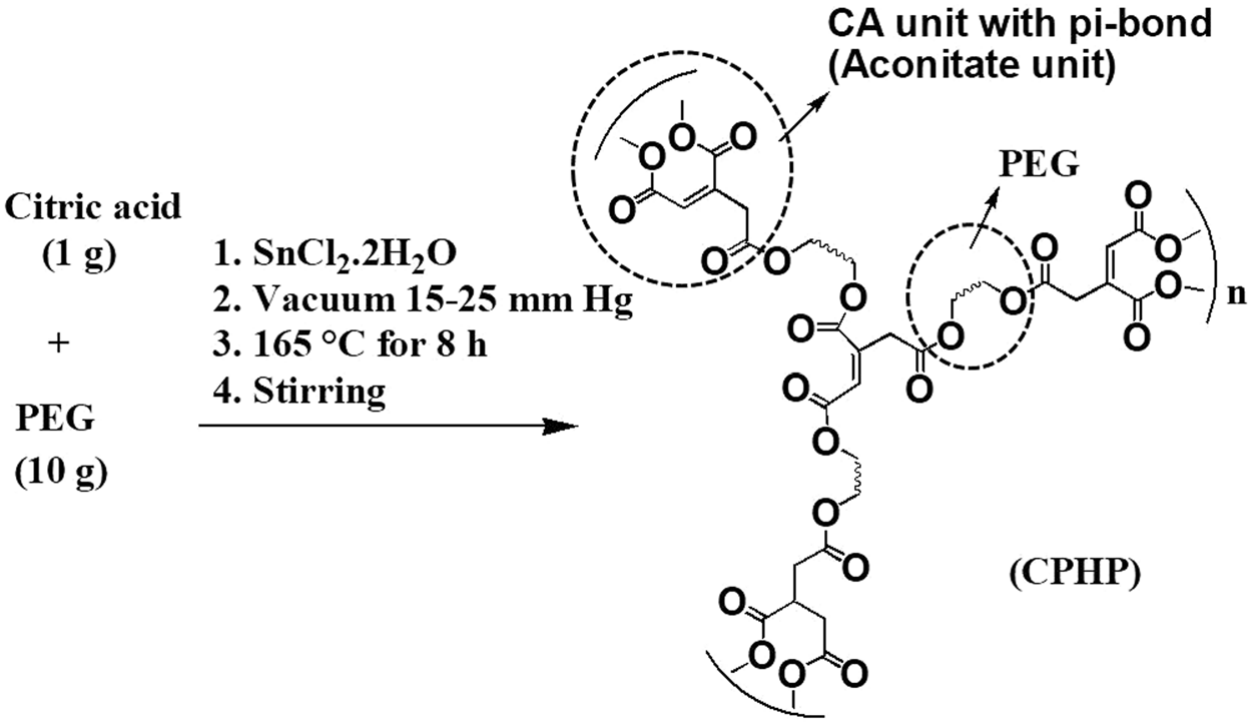
Synthesis of CPHP.

CPHP was synthesized by direct melt polycondensation under vacuum as shown in Fig. 1. When the reaction was carried out at 165 °C, citric acid and PEG melted initially to give a colorless transparent viscous liquid which subsequently underwent polymerization. The colorless transparent viscous liquid became dark brown viscous liquid and finally a dark brown colored solid sheet like layer formed after completion of the reaction. The sheet like solid product was dissolved in hot chloroform and precipitated as a white powder (mp = 42 °C) by the addition of ice cold diethyl ether. The structure of CPHP and the presence of a π-bond in the polymer backbone were confirmed by spectral studies (Fig. 1).

In the FTIR spectrum (Fig. S1), a broad band in the range of 3300–3600 cm^−1^ corresponds to the –OH stretching frequency while the stretching frequency of –CH_2_ group appears in the region 2879–2909 cm^−1^. The peaks at 1642 cm^−1^ and 1722 cm^−1^ are due to the stretching vibrations of C=C and the –C=O group of α, β unsaturated ester respectively^24^. The stretching frequency of –C–O group appears at 1102 cm^−1^. In the ^1^H-NMR spectrum (Fig. S2), two proton signals corresponding to the –CH_2_ groups of citrate and PEG are found at 2.8 ppm and 3.6 ppm respectively and methine proton signal of the cis- and trans-aconitate units appear at 5.6 and 6.2 ppm respectively. The ^13^C-NMR spectrum exhibits eight signals (Fig. S3). The signal at 175 ppm (a) is assigned to the carbonyl carbon of the free carboxylic acid group, where as the two signals at 169 ppm (b) and 165 ppm (c) are assigned to the carbonyl carbon of the saturated ester and unsaturated ester groups respectively^25–27^. The signals at 132 ppm (d) and 128 ppm (e) are assigned to R_2_C=CHR carbons, where as the two signals at 37 ppm (f) and 43 ppm (g) are assigned to methylene carbons of the citrate units^25–27^. The methylene carbons of PEG appear as a sharp peak at 70 ppm (h), while the alcoholic carbon of the free citric acid group merges with the signal (h). A peak at 87.4 ppm is due to the methylene protons of PEG which is connected to the citrate unit. The FTIR and NMR spectral results confirm the presence of R_2_C=CHR, –OH, –COOH, –CH_2_ and saturated and unsaturated ester groups in the CPHP molecule and they also indicate that the –OH group and methylene proton of citric acid are partly involved in the condensation of water molecule to produce aconitate units. The molecular weight of CPHP determined by MALDI-TOF mass spectrum is 195515 (g/mol) (Fig. S4).

Namazi *et al*. first reported the synthesis of citric acid-PEG (CA-PEG) dendrimer by the solution phase esterification method^11^ whereas Naeini *et al*. synthesized CA-PEG dendrimer by direct melt polycondensation technique^13^. The PEG1500 was employed as a core unit while CA was involved in building up the backbone of CA-PEG linear dendrimer. CA and PEG1500 with CA/PEG ratio of 2, 5, 8 and 10 have been employed in the direct melt polycondensation and a low molecular weight (3800 g/mol). CA-PEG dendrimer was obtained without any π-bond in the back bone of the dendrimer. In the present investigation, the ratio of CA/PEG was found to be 1/10 in the direct melt polycondensation of CA and PEG6000 and the reaction yielded CPHP with high molecular weight with π-bond in the polymer backbone.

The above results suggest that when the reaction is carried out with a higher amount of CA, CA predominates in building the repeated generations of the dendrimer backbone due to its greater availability resulting in linear dendrimer. Since, the -OH group of CA is involved in condensation with the –COOH group of CA of another generation, CA-PEG linear dendrimer does not have a π-bond. In the present study, when the reaction is carried out with a higher amount of PEG, PEG acts as a linking agent in connecting CA units of two different molecules resulting in CPHP and the hydroxyl group of some CA condense with the neighboring methylene protons resulting in the formation of π-bond in the polymer backbone (aconitate units) (Fig. 1).

**Figure 2:**
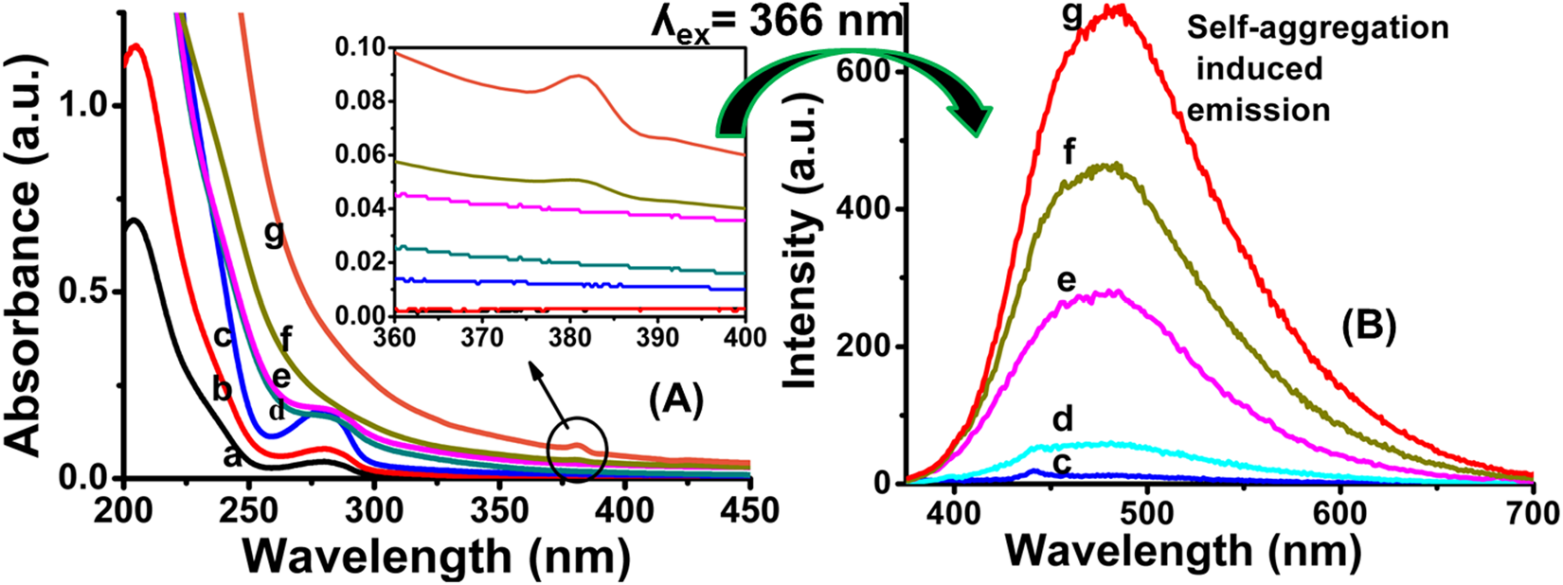
(**A**) UV-Visible absorption and (**B**) Emission spectra of CPHP (λ_ex_ = 366 nm) with concentration of CPHP (a) 1.25 × 10^−6^ g/mL, (b) 5 × 10^−6^ g/mL, (c) 5 × 10^−5^ g/mL, (d) 5 × 10^−4^ g/mL, (e) 5 × 10^−3^ g/mL, (f) 1.25 × 10^−2^ g/mL and (g) 2.5 × 10^−2^ g/mL.

The UV-Visible spectrum of CPHP exhibits maximum absorption at 279 nm due to π-π* transition (Fig. 2A). The UV-Visible absorption spectrum of CPHP recorded in the concentration range of 1.25 × 10^−6^ g/mL–2.5 × 10^−2^ g/mL indicates that the absorbance value increases with an increase in concentration up to 5 × 10^−5^ g/mL. When the concentration is increased from 5 × 10^−4^ g/mL to 5 × 10^−3^ g/mL, the absorption peak is broadened and further increase in the concentration from 1.25 × 10^−2^ g/mL to 2.5 × 10^−2^ g/mL, the peak at 279 nm becomes broader and a new peak appears at 380 nm due to intermolecular π_(c=c)_-σ*_(CH)_ transition (inset in Fig. 2A). The aggregation of CPHP with respect to its concentration can be understood from the plot of concentration Vs. absorbance. A linear absorbance profile is observed up to 5 × 10^−5^ g/mL of concentration and above which the linearity deviates drastically (Fig. S5). The line broadening and non-linear absorbance profile in the UV-Visible absorption spectra can be attributed to self-aggregation of CPHP above the concentration 5 × 10^−5^ g/mL. Similar observation on self-aggregation of organic molecules and polymers were confirmed by spectral analyses in the literature^18,28^.

**Figure 3:**
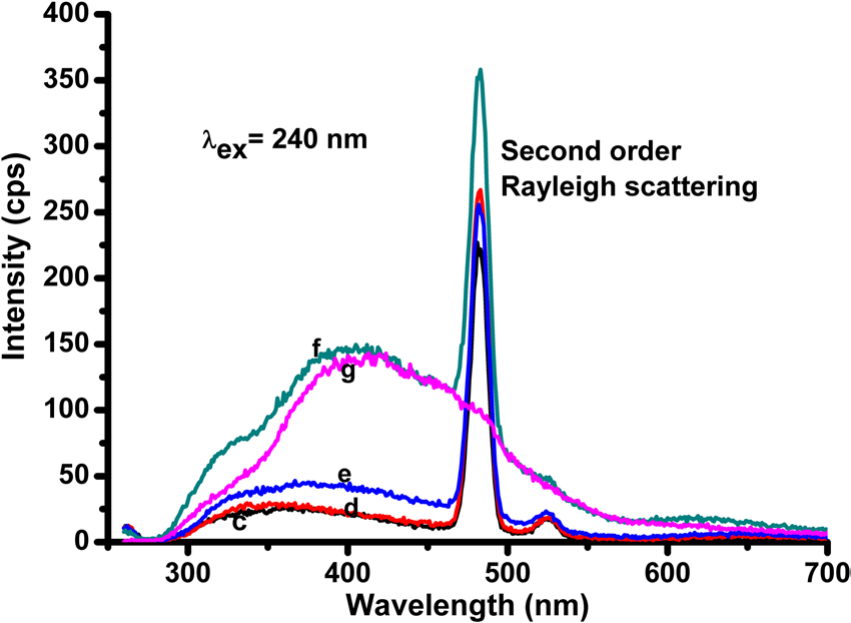
UV-Visible emission spectra of CPHP (λ_ex_ = 240 nm) at (c) 5 × 10^−5^ g/mL, (d) 5 × 10^−4^ g/mL, (e) 5 × 10^−3^ g/mL, (f) 1.25 × 10^−2^ g/mL and (g) 2.5 × 10^−2^ g/mL concentration of CPHP.

The emission spectra of CPHP were recorded by exciting at 240 nm and compared with those of CPHP recorded by exciting at a slightly higher energy than the new absorption signal (366 nm). The emission spectra of CPHP recorded by exciting at 240 nm exhibit a broad emission peak from 300 nm to 700 nm associated with a second order Raleigh scattering at 480 nm (Fig. 3). The Raleigh scattering could be characterized by disappearance of the sharp signal at 480 nm after increasing the concentration to 2.5 × 10^−2^ g/mL (Fig. 3). Since, the excitation of CPHP molecule was made possible by absorption of photons at 380 nm, the emission spectra were recorded by exciting at 366 nm (Fig. 2B). Interestingly, the clear emission spectra are obtained by exciting at 366 nm and they exhibit a broad emission peak between 390 and 700 nm without any scattering signal (Fig. 2B). This observation suggests that the photon emission process is only due to the absorption of light at 380 nm and not due to the absorption of light at 240 nm. Since, the absorption of light at 380 nm is possible only by the aggregation of CPHP inferred from the UV-Visible spectral results (Fig. 2A), the emission process is identified as an AIE. Further, emission intensity increases with a bathochromic shift when the concentration is increased. suggesting the SAIE of CPHP^28^.

**Figure 4:**
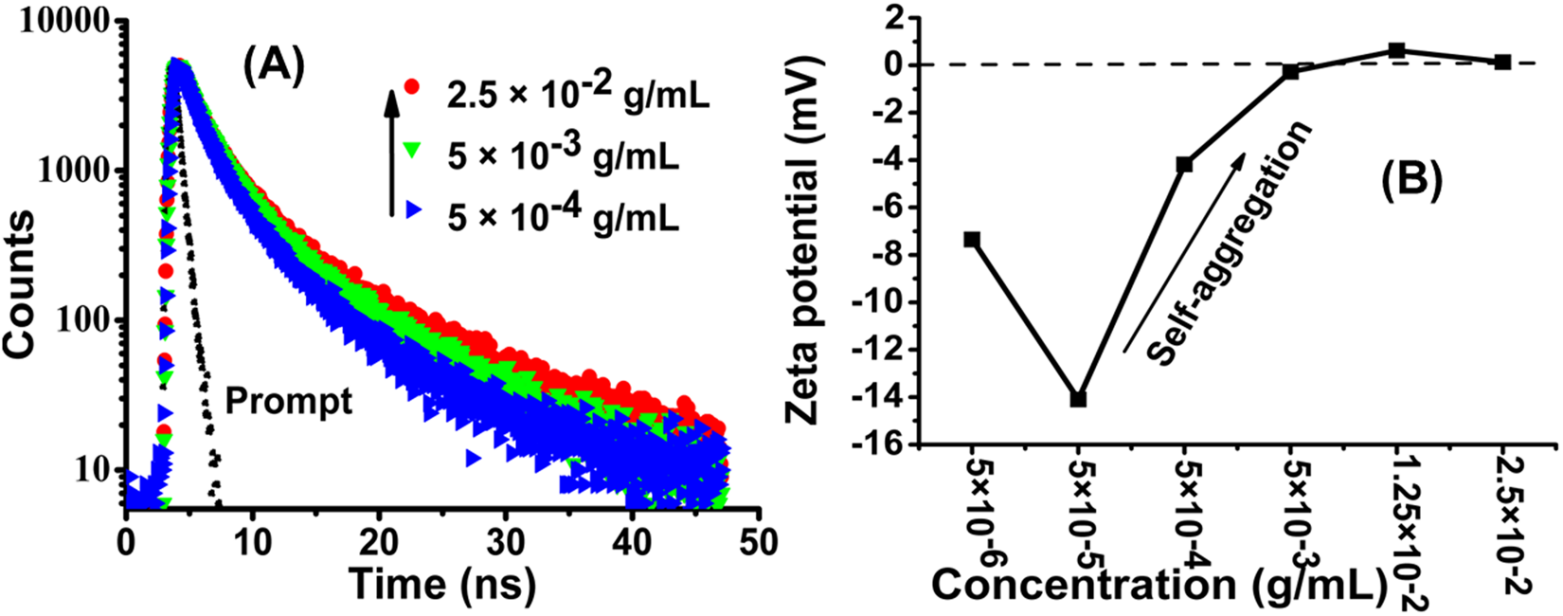
(**A**) Fluorescence decay curves of CPHP, (**B**) plot of zeta potential Vs concentration.

The fluorescence quantum yield (Φ_f_) of CPHP measured against quinine sulfate also increases from 0.019–0.29 with an increase in concentration (Table S1) which is contrary to the usual emission phenomenon of fluorophores indicating the SAIE of CPHP. The fluorescence life time (τ_f_) by TCSPC measurements also support the SAIE property of CPHP. The fluorescence decay of CPHP at three different concentrations is shown in (Fig. 4A)and the τ_f_ values are tabulated (Table S2). The fluorescence decay exhibits a tri-exponential fit, which is a characteristic property of aggregates of polymers^29^, thereby confirming the formation of aggregates of CPHP due to self-organization. The τ_f_ is independent of concentration, whereas the τ_f_ (for example T3) of the CPHP molecule increases from 4.93 ns to 11.38 ns with an increase in concentration from 5 × 10^−4^ g/mL to 2.5 × 10^−2^ g/mL indicating the SAIE of the CPHP molecule (Table S2)^30^. The CPHP exhibits longer excited state life time in the aggregated state (11.38 ns), when compared to that of the self-aggregation induced green emission of the reported systems (1.15 ns – 6.6 ns)^30–32^.

**Figure 5:**
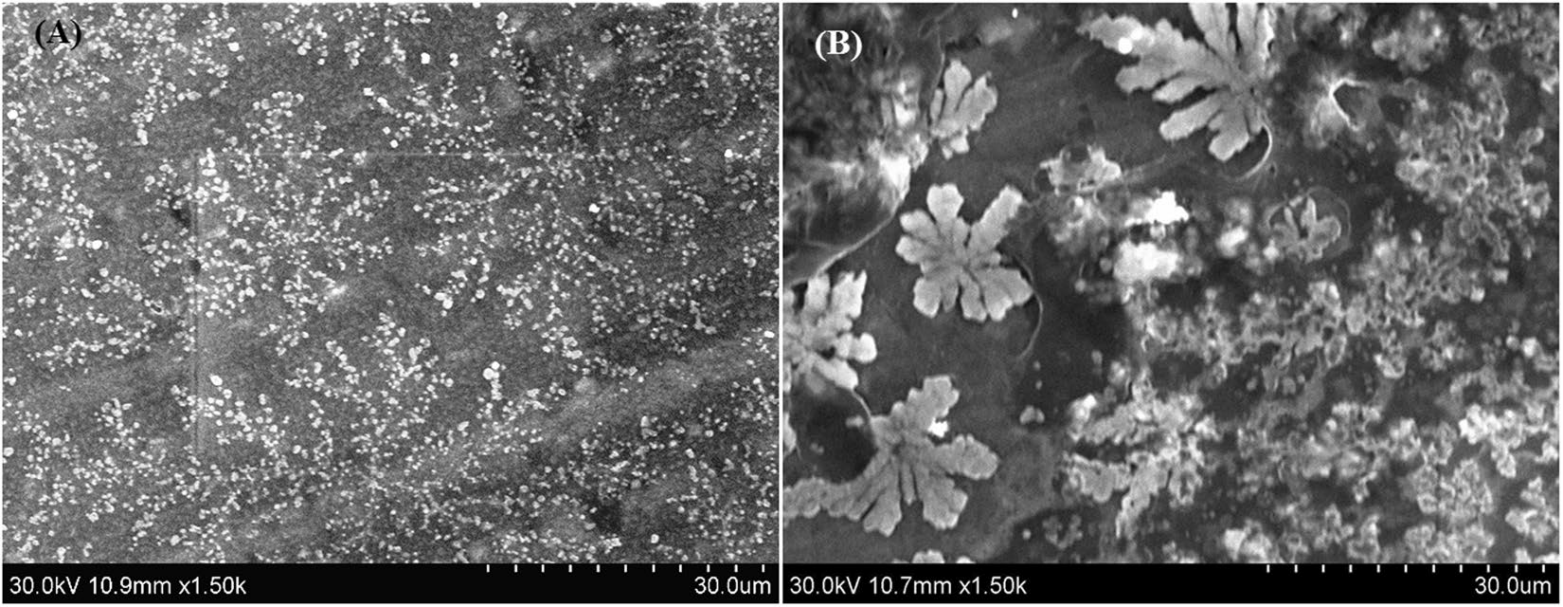
SEM images of CPHP at the concentration of (**A**) 5 × 10^−3^ (g/mL) and (**B**) 2.5 × 10^−2^ (g/mL).

The zeta potential values with increasing concentration of CPHP are given in Fig. 4B. The negative zeta potential for the highly diluted solution of CPHP is due to the presence of free carboxylic acid groups. When the concentration is increased from 5 × 10^−6^ g/mL to 5 × 10^−5^ g/mL, the zeta potential is lowered from −7.35 mV to −14.1 mV which is attributed to an increase in the free carboxylate groups. When the concentration is further increased to 5 × 10^−4^ g/mL and 5 × 10^−3^ g/mL, the zeta potential changes in the positive direction to −4 mV and −0.278 mV respectively. Before the aggregation point (5 × 10^−3^ g/mL), free carboxylate anions are present and hence a negative zeta potential is observed. After the aggregation point, the carboxylate anions are neutralized by the formation of weak hydrogen bond or CH/CO interaction resulting in the reversal of zeta potential towards the positive direction^33^. These results confirm the self-aggregation of CPHP molecule above the concentration of 5 × 10^−5^ g/mL. The self-assembled CPHP molecule with different concentrations has been coated on an aluminium foil and the morphology is investigated by SEM after drying under vacuum (Fig. 5). It is observed that the CPHP molecule assembles in a uniform fashion to exhibit a branched leaf like structure due to self-aggregation (Fig. 5).

It has widely been reported that AIE takes place in a mixture of solvents. The compound containing organic fluorophores are usually soluble in organic solvents such as tetrahydrofuran (THF), chloroform (CHCl_3_), acetonitrile and ethanol, while water or methanol is added as a non-solvent during which an aqueous suspension of organic nanoparticles are formed via a mild solvent exchange precipitation method^30,34–36^. Interestingly, AIE can also be achieved by increasing the viscosity of the solution^37^. In the present study, the CPHP is an amphiphilic molecule and exhibits SAIE property in 100% aqueous medium. A series of aqueous CPHP solutions with different concentrations was prepared and examined for AIE by UV-Visible absorption spectral, fluorescence spectral, zeta potential and time correlated life time measurements. The results confirm the SAIE property of CPHP in 100% aqueous medium and it does not require any non-solvent. The aggregation of CPHP occurred by simple intermolecular hydrogen bonding and the aggregates are also soluble in water due to the presence of PEG. Hence, the CPHP molecule exhibits SAIE property with an opposite polarity to that of other systems reported earlier^19^.

Recently, Tang’s group has extensively studied the mechanism of AIE of various materials such as substituted ethylene systems and polypeptides^37–39^. Several mechanisms have been reported for AIE such as restricted intramolecular rotation (RIR), restricted intramolecular vibration, restricted intramolecular motion and extremely fast excited state intramolecular proton transfer (ESIPT)^37,38^. Since, two types of excitations occur in the UV-visible absorption spectrum of CPHP aggregates, it is important to identify the correct excitation type responsible for SAIE. To investigate the mechanism of SAIE of CPHP, the reported CA-PEG linear polymer without a π-bond (aconitate unit) was also synthesized by following the literature procedure^13^ and it did not exhibit any luminescence property (data not shown). Since CA-PEG linear polymer also contains carboxylate group, SAIE may not be due to CH/CO interaction (hydrogen bonding with the carboxylate groups) and hence the absorption at 380 nm is not due to n - π* transition. If π - π* transition is the reason for SAIE of CPHP aggregates, the emission signal could not have been observed when the molecule was excited at 380 nm corresponding to π(c = c) - σ*(CH) transition. Since, the excitation process could be achieved by a lower energy at 366 nm, the emission process could be favored by the π(c = c) - σ*(CH) transition and not by the π - π* transition (Fig. 2). The absorption of light at 380 nm due to the π_(C=C)_ - σ*_(C-H)_ transition promote the electrons to the excited state, and CPHP aggregates exhibited SAIE due to the restricted intramolecular vibration (or) restricted intramolecular rotation in the aggregated state. The π-bond in the aconitate unit interacts with the CH bond of PEG or CA units which induces the green emission where as PEG moiety in the CPHP induces the solubility.

**Figure 6:**
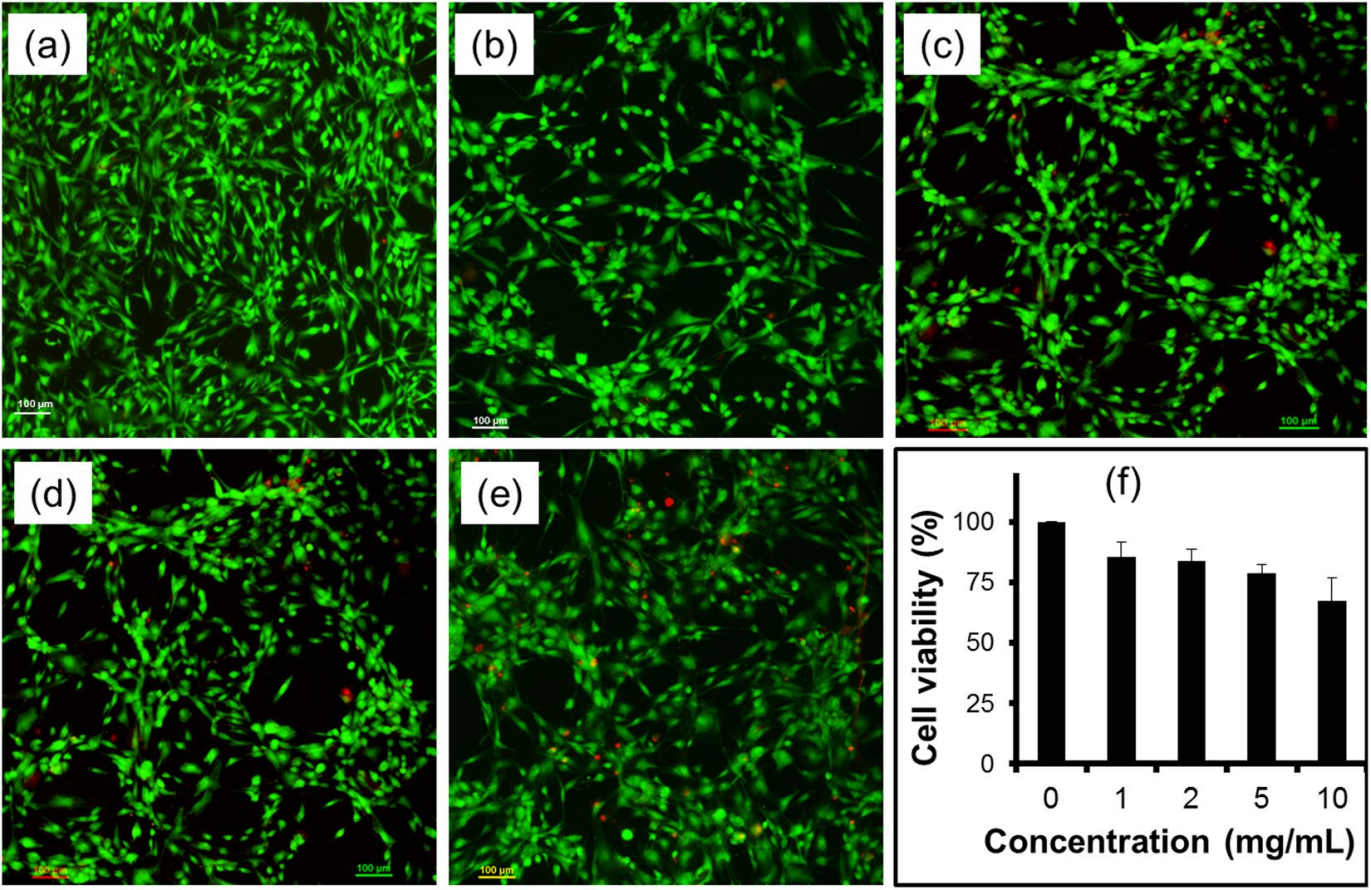
*In vitro* cytotoxicity results. (**a**–**e**) Fluorescent micrographs of live and dead cell assay: (**a**) control, (**b**) 1 mg, (**c**) 2 mg, (**d**) 5 mg and (**e**) 10 mg of CPHP, and (**f**) WST-1 assay.

The possibility of utilizing this CPHP molecule in various biomedical applications such as drug delivery and tissue engineering has been emphasized by investigating its biocompatibility in adipose- derived stem cells (ADSCs) (vide: supporting information). The *in vitro* cytotoxicity test results show that the CPHP resulted in higher than 80% of cell viability up to a concentration of 5 mg/mL (Fig. 6f). This concentration level is highly prominent when compared to the cell viability concentrations of the recently reported materials used for biomedical applications^40–42^. Fluorescence cell line images also indicate that the majority of CPHP- treated ADSCs resemble the morphology of control cells with green fluorescence (Fig. 6a–e). These results suggest that the CPHP molecule is highly biocompatible even at higher concentration towards ADSCs. Hence, it can be utilized as an extracellular matrix for tissue engineering applications.

In summary, the present investigation provides an interesting green approach for the solvent- free synthesis of citric acid-PEG branched polymer. The water soluble self-aggregates induced green emission of CPHP has been demonstrated by zeta potential measurement, UV-Visible absorption and emission spectral studies. The biocompatible CPHP may find potential application in various biomedical fields.

## Methods

### Synthesis of CPHP

1 g of citric acid, 10 g of PEG6000 and 0.11 g of stannous chloride dihydrate were placed in a R.B. flask equipped with a distillation bridge and a magnetic stirrer. The reaction mixture was initially heated to melt after which the reaction was carried out at 165 °C under high vacuum (15–25 mm Hg) for 8 h. Then it was cooled to room temperature, dissolved in hot chloroform and precipitated by the addition of ice-cold diethyl ether. The white powder was washed three times with ice-cold diethyl ether and dried under vacuum. (mp = 42 °C, molecular weight = 195515 g/mol).

### UV-Visible absorption spectral analyses

The CPHP solution was prepared in Millipore water at various concentrations (1.25 × 10^−6^ g/mL − 2.5 × 10^−2^ g/mL) and UV-Visible absorption spectral analyses were carried out on a Shimadzu (UV-1800) UV-Visible spectrophotometer.

### Photo luminescence spectral analyses

The CPHP solution was prepared in Millipore water at various concentrations (1.25 × 10^−6^ g/mL − 2.5 × 10^−2^ g/mL). The photoluminescence spectral analyses were carried out on a Cary Eclipse fluorescence spectrophotometer. The excitation wavelength was set to 366 nm to record the fluorescence spectrum of CPHP.

### Time- correlated single photon counts (TCSPC)

Time-resolved fluorescence decays were obtained by the time-correlated single photon counting (TCSPC) technique. A Jobin-Yvon IBH LED was used as an excitation source to excite at 375 nm. The fluorescence emission at the magic angle (54.78) was counted at 480 nm by an MCP PMT apparatus (Hamamatsu R3809U). The instrument response function was 52 ps. The fluorescence decay was analyzed by using IBH (DAS 6) (UK) software and the data were fitted to multi exponential fit.

## Electronic supplementary material

Supporting information
