# Supplementary material for: Water Soluble Self-Aggregates Induced Green Emission of Biocompatible Citric Acid-PEG Hyper Branched Polymer
Source: Sci Rep. 2017 Nov 27;7:16418. doi: 10.1038/s41598-017-16683-w (PMC5704015; doi:10.1038/s41598-017-16683-w)
Supplement: Supplementary file 1 — Supporting information [file 41598_2017_16683_MOESM1_ESM.doc]

# **Water Soluble Self-Aggregates Induced Green Emission of Biocompatible Citric Acid-PEG Hyper Branched Polymer**

# **Mani Gajendiran^1,2^, Kyobum Kim^2*^, Sengottuvelan Balasubramanian^1,3*^**

*^1^Department of Inorganic Chemistry, Guindy Campus, University of Madras, Chennai - 600025, India.*

*^2^Division of Bioengineering, College of Life Sciences and Bioengineering, Incheon National University, Incheon, Korea.*

^3^Center for Advanced Materials Research, Vels University, Chennai-600117, India.

^1^Corresponding Author E-mail:

Prof. Dr. S. Balasubramanian : [bala2010@gmail.com](mailto:bala2010@gmail.com)

^2^Co-corresponding author:

Prof. Dr. Kyobum Kim: [kyobum.kim@inu.ac.kr](mailto:kyobum.kim@inu.ac.kr)

**Supporting information**

1. **EXPERIMENTAL**
   1. **Materials**

Citric acid (Sigma Aldrich), PEG6000 (HiMedea), Stannous chloride dihydrate (Fischer Scientific Co) and all solvents used were of HPLC grade.

- 1. **Characterization techniques**
     1. **FTIR spectrum**

The FTIR spectrum of the CPHP was recorded with the KBr pellet on a Perkin-Elmer 8300 FTIR spectrometer.

- - 1. **^1^H-NMR spectrum**

^1^H-NMR spectrum was recorded on a Bruker 300 MHz NMR spectrometer using CDCl_3_ as a solvent and tetramethylsilane (TMS) as the internal standard.

- - 1. **^13^C-NMR spectrum**

The ^13^C-NMR spectrum was recorded on a Bruker 400MHz NMR spectrometer in CDCl_3_.

**1.2.4. MALDI-TOF mass spectrum**

The MALDI-TOF mass spectral study was conducted on a Voyager DE PRO Biospectrometry Workstation MALDI TOF MS instrument. A pulsed nitrogen laser of
337 nm was used for desorption ionization and the measurement was carried out in the linear mode.

**1.2.5. SEM images**

The SEM images were captured by using a Hitachi S-3000H scanning electron microscope. The CPHP was dissolved in water and coated on aluminium foil by drop casting. It was dried under vacuum overnight before analysing by SEM.

**1.2.6. Zeta potential**

The CPHP was prepared in Millipore water at various concentrations
(1.25 × 10^-6^ g/mL - 2.5 × 10^-2^ g/mL) and zeta potential measurements were carried out on a Malvern Zetasizer (Nano ZS) instrument.

**1.3. *In vitro* cytotoxicity**

**1.3.1. Cell culture**

The growth media was prepared with Dulbecco’s modified Eagle’s medium (DMEM) (WISENT Inc.) (89 % (v/v)), fetal bovine serum (FBS) (Corning) (10 % (v/v)), and Penicillin-Streptomycin solution (WISENT Inc.) (1 %). The experimental wells were designed in to 5 groups in a 24 well cell culture plate (each groups in four replicate wells). To group-1, only growth media was added and used as control group. 1 mg, 2 mg, 5 mg and 10 mg of CPHP were weighed and added to group 2, group 3, group 4 and group 5 wells respectively. The adipose derived stem cells (ADSC) were added to all the experimental wells with a cell count of 80000 cells per well, and incubated at 37 ℃ with 5 % CO_2_ and 95 % of humidity for 24 h. After 24 h of incubation, the cell viability was investigated by WST 1 assay ^S1^ and *live and dead cell* (L/D) assay methods.

**1.3.2. WST 1 assay**

Cell proliferation assay was carried out using WST-1 assay kit (EZ-Cytox cell viability assay kit, DAEIL Lab Service Co. Ltd). The WST 1 assay solution and growth media (DMEM 89 %, FBS 10 % and Streptomycin-Penicillin 1 %) were mixed in the volume ratio of 1:10. The ADSC cells (80000 cells/per well) were cultured in 24 well plate in growth media and incubated at 37 ℃ for 24 h. After incubation of 24 h, the growth media were removed from the well, and the cells were washed twice with Dulbecco’s phosphate buffer saline 1x (WISENT Inc.) (PBS). The growth media contained WST 1 assay solution (400 μL) was added to the wells and incubated at 37 ℃ for 3 h under dark. After 3h, the supernatant (100 μL) was transferred to another 96 well plate and the optical density of the solution was measured at 440 nm by using UV spectrophotometer (Thermo scientific). The cell viability was calculated as follows,

Cell viability = (OD of sample/OD of control)*100

**1.3.3. Live and dead cell assay**

The live and dead cell (L/D) assay solution was prepared by mixing 0.5 µL of Calcein-AM (4 mM) (Thermo scientific) and 2 µL of Ethidium homodimer-1 (2 mM) (Thermo scientific) in 997.5 µL of PBS (pH 7.4). After 24 of incubation, the media was removed from cell cultured wells and the ADSC cells were washed twice with PBS. Then, L/D assay solution (100 μL) was added to each well and incubated at 37 °C for 30 minutes under dark. The stained cells were observed by fluorescent microscope (Nikon).

**2. Results**

**2.1. Characterization of CPHP**

#
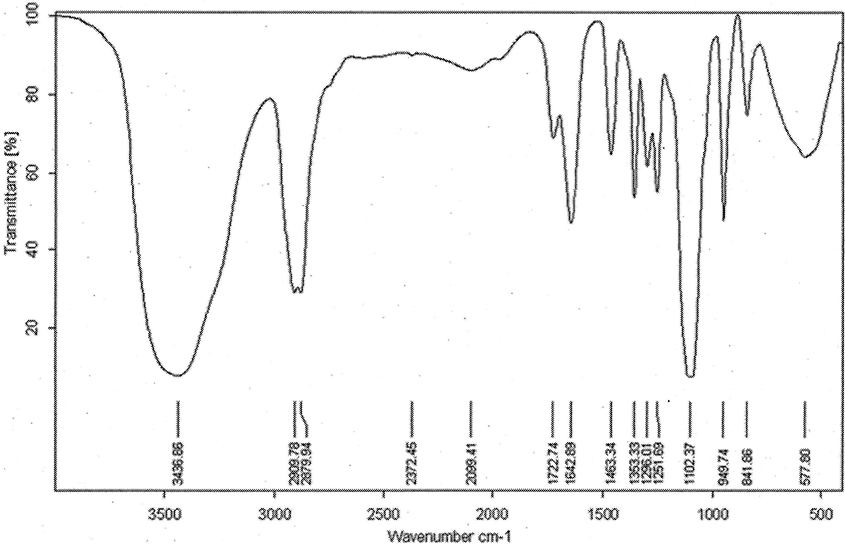


**Figure S1.** FTIR spectrum of CPHP.


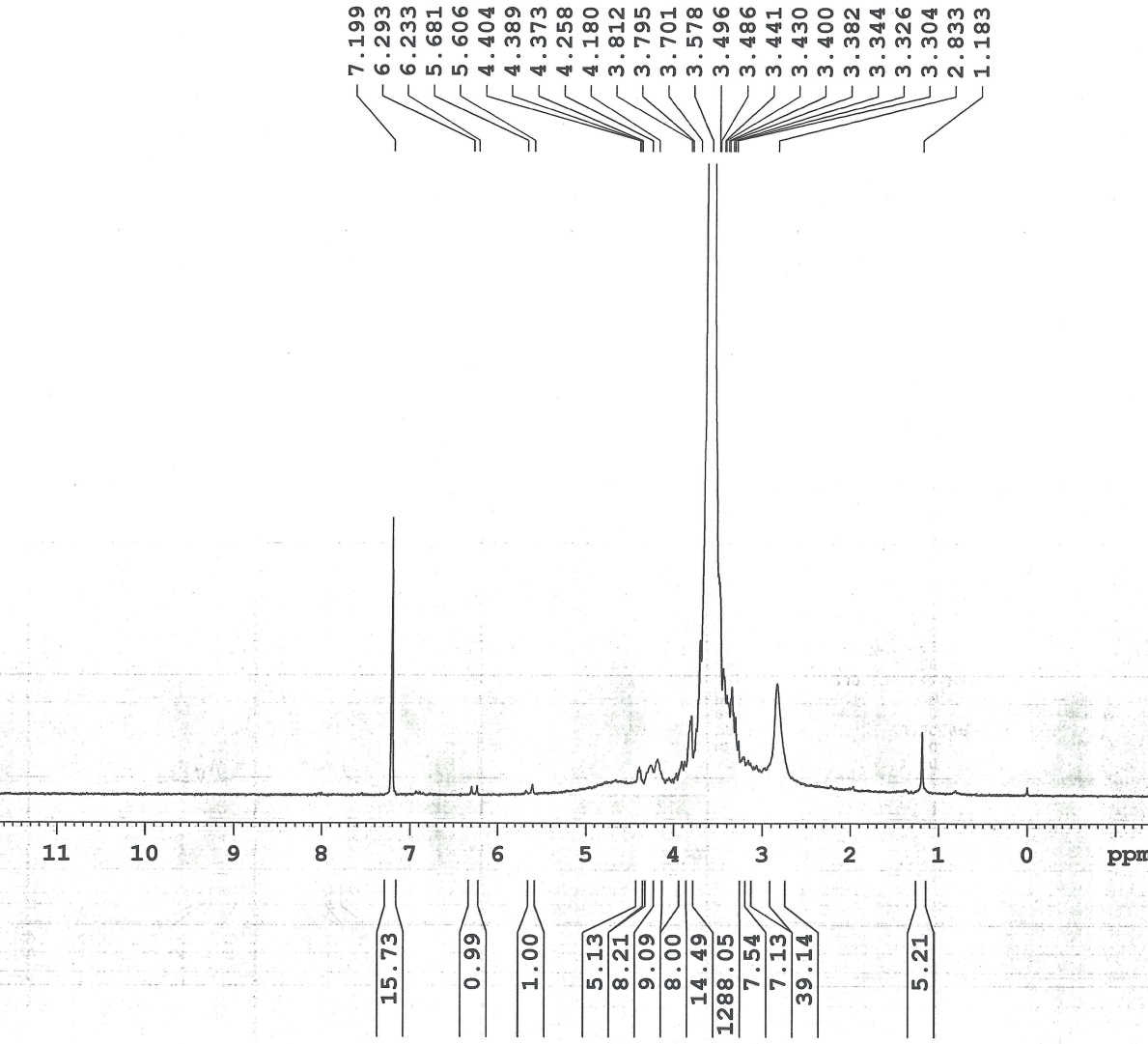


**Figure S2.** ^1^H-NMR spectrum of CPHP in CDCl_3_.

**Note:**

(Since, the PEG content is much higher than the citrate moiety in the CPHP, the proton signal corresponding to CH_2_ group of PEG appears as a strong peak at 3.6 ppm when compared to the signals of citric acid. It can be understood by noticing the week intensity of TMS peak at 0.0 ppm. Since the overall percentage of citrate moiety is calculated to be 3.2 % and, out of this 3.2 %, only 0.61 % of aconitate units are present in the CPHP molecule. Hence, the small peaks at 5.6 ppm and 6.2 ppm corresponding to the methine proton signal of cis- and trans-aconitate are very week due to the lesser composition of aconitate unit).


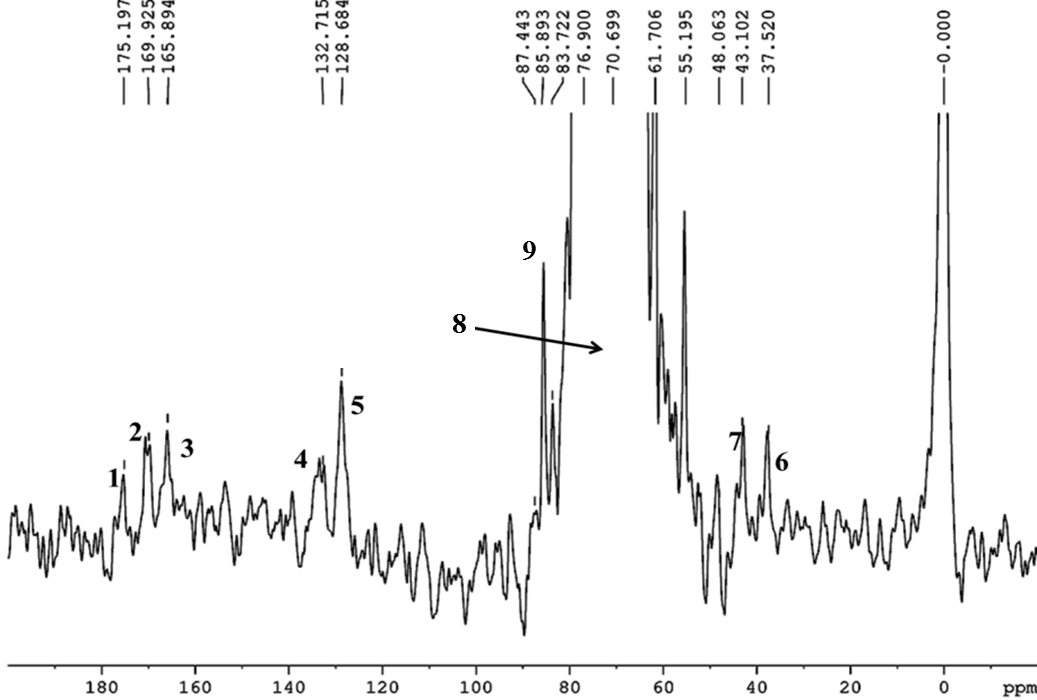


**Figure S3.** ^13^C-NMR spectrum of CPHP in CDCl_3_ (Number of scans = 7200).

(Since, the PEG content is much higher in the CPHP, the CH_2_ signal (8) of PEG appears as a strong peak compared to the signals of CA)


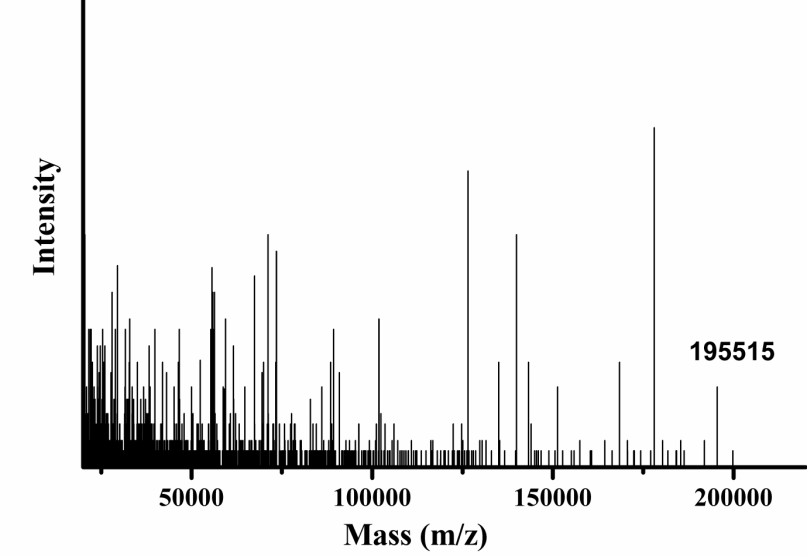


**Figure S4.** MALDI-TOF mass spectrum of CPHD.


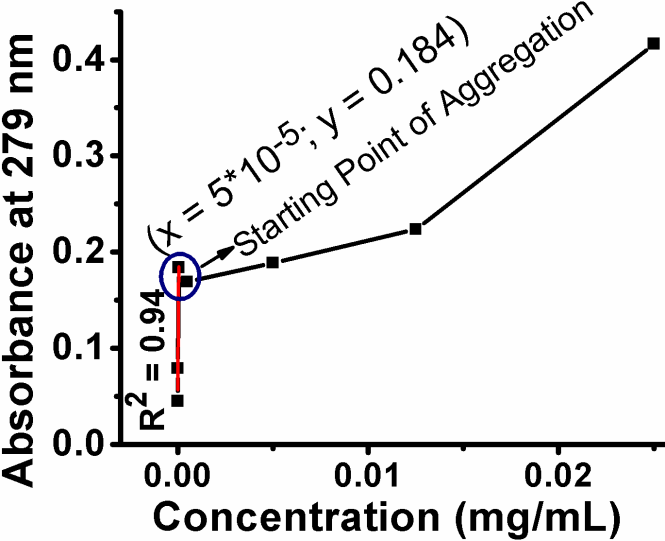


**Figure S5.** Plot of absorbance at 279 nm Vs. concentration of CPHP.

**2.2. Determination of fluorescence quantum yield of CPHP with different concentrations**


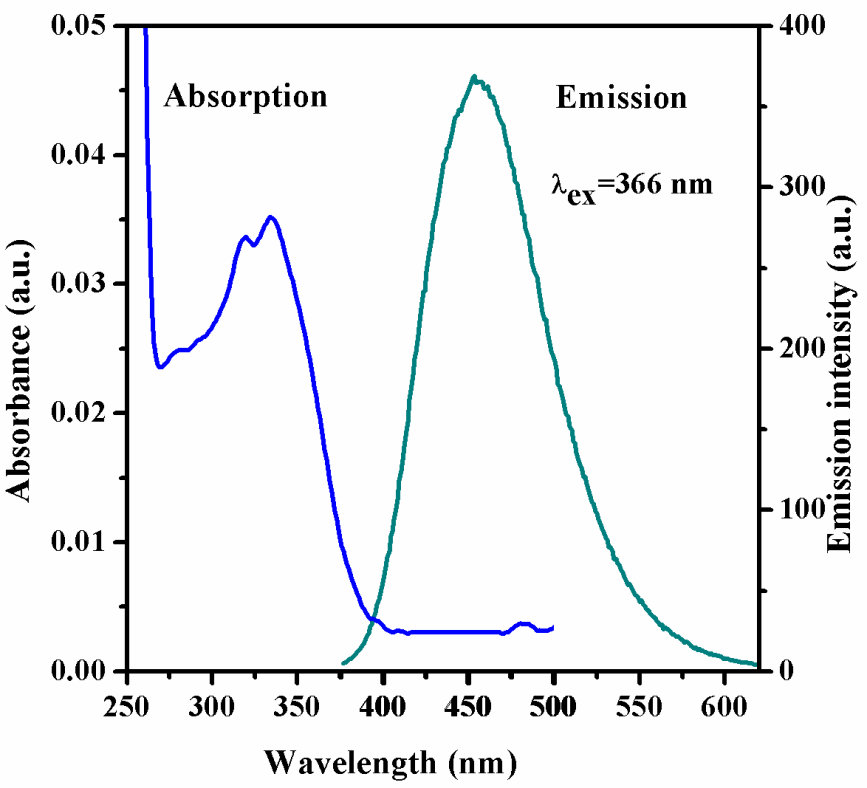


**Figure S6.** UV-Visible absorption and emission spectra of quinine sulphate in 0.05 N H_2_SO_4_ recorded as a standard for quantum yield calculation of CPHP.

Table S1. Fluorescence quantum yield of CPHP at different concentrations

| **CPHP**  **Concentration (g/mL)** | **Quantum yield against**  **Quinine sulphate** |
| --- | --- |
| 5 × 10^-5^ | 0.01948 |
| 5 × 10^-4^ | 0.0836 |
| 5 × 10^-3^ | 0.2195 |
| 1.25 × 10^-2^ | 0.29098 |
| 2.5 × 10^-2^ | 0.29344 |

Since, the emission is caused by the absorption of light at 370 nm, the excitation wavelength was fixed at slightly above the absorption maximum (366 nm). The fluorescence quantum yield of the CPHP with different concentration was determined against quinine sulphate by exciting at 366 nm. Emission quantum yield of quinine sulphate is 0.53 ^S2^.

The fluorescence quantum yield of CPHP was calculated by using the following formula,

Φ_(s)_ = [I_(s)_×A_(r)_×Φ_(r)_]/[I_(r)_ ×A_(s)_]

Where, Φ_(s)_ - Quantum yield of sample

I_(s)_ - Emission intensity of sample

A_(s)_ - Absorbance of sample

Φ_(r)_ - Quantum yield of reference

I_(s)_ - Emission intensity of reference

A_(s)_ - Absorbance of reference

**2.3. Time correlated single photon counts (TCSPC)**

The fluorescence decay curves are fitted to a tri-exponential model.

Table S2. Fluorescence life time (τ) of CPHP molecule at different concentrations

| **Concentration**  **(g/mL)** | **Life time (τ) and Amplitude** | | | | | | |
| --- | --- | --- | --- | --- | --- | --- | --- |
|  | **T1 (s)** | **Amplitude (%)** | **T2 (s)** | **Amplitude (%)** | **T3 (s)** | **Amplitude (%)** | **χ^2^** |
| 5 × 10^-4^ | 7.72 × 10^-11^ | 16.3 | 1.51 × 10^-9^ | 35.7 | 4.93 × 10^-9^ | 49.8 | 1.1 |
| 5 × 10^-3^ | 3.91 × 10^-12^ | 9.98 | 1.93 × 10^-9^ | 30.1 | 6.93 × 10^-9^ | 60.01 | 1.0 |
| 2.5 × 10^-2^ | 1.14 × 10^-9^ | 5.18 | 3.28 × 10^-9^ | 21.91 | 11.38 × 10^-9^ | 72.91 | 1.0 |

**References**

(S1) Blanch, A. R.; Méndez, J.; Castel, S.; Reina, M. *J. Microbiol. Methods* **2014**, *103*, 64.

(S2) A. M. Brouwer, *Pure Appl. Chem.* **2011**, *83,* 2213.
